# Association between combined occupational exposure to coal dust and noise and high-frequency hearing loss among workers: a cross-sectional study

**DOI:** 10.3389/fpubh.2026.1929586

**Published:** 2026-09-04

**Authors:** Jiaqi Ma, Yonghu He, Jie Meng, Bicheng Li, Rongmei Song, Baoheng Liu, Dandan He, Ye Ruan

**Affiliations:** 1 School of Public Health, Lanzhou University, Lanzhou, China; 2 Baiyin City Center for Disease Control and Prevention, Baiyin, China; 3 China Railway Lanzhou Group Co., Ltd., Lanzhou, China

**Keywords:** coal dust, combined exposure, high-frequency hearing loss, noise exposure, occupational expossure

## Abstract

**Background:**

Occupational environments contain a wide variety of harmful factors, with noise often coexisting with other physical, chemical factors, leading to combined exposure that may increase the risk of hearing impairment among workers. This study aims to investigate the association of occupational noise and coal dust combined exposure on noise-induced hearing loss in workers, providing evidence for the prevention and control of occupational noise-induced hearing loss in occupational populations.

**Methods:**

We conducted a cross-sectional study of 1,187 occupationally exposed workers. These workers who underwent occupational health examinations at a certain occupational health examination institution in Baiyin City in 2023 were selected as study subjects and divided into a noise only group (276 participants) and a combined noise-coal dust exposure group (911 participants). Pure-tone air-conduction audiometry was used to assess hearing loss, and the *χ*^2^ test and multivariate logistic regression analysis were applied to identify risk factors for high-frequency hearing loss (HFHL).

**Results:**

The prevalence rates of HFHL were 35.5 and 46.8% in the noise group and the combined noise-coal dust exposure group, respectively, with a statistically significant difference (*p* < 0.001); the prevalence of speech-frequency hearing loss was relatively low. Hearing abnormalities were primarily concentrated in the high-frequency range, with a marked decline around 4,000 Hz. Multivariate logistic regression analysis indicated that age, gender, and exposure to occupational hazards were independent risk factors for HFHL. After multivariable adjustment, combined exposure to occupational noise and coal dust was associated with higher odds of HFHL compared with noise exposure alone (OR = 1.620, 95% CI: 1.198–2.190, *p* = 0.002).

**Conclusion:**

Occupational noise-induced hearing loss is predominantly HFHL, and Workers classified as jointly exposed to occupational noise and coal dust had higher adjusted odds of HFHL than workers classified as exposed to occupational noise alone. Comprehensive protection and occupational health surveillance for workers exposed to combined hazards should be strengthened.

## 1 Introduction

Hearing loss is a major global public health problem and currently ranks as the third leading cause of years lived with disability worldwide (1). Occupational noise exposure is an important cause of adult hearing loss, accounting for approximately 16% of cases, and occupational noise-induced hearing loss (ONIHL) is now one of the most common occupational diseases globally, especially in high-noise industries such as manufacturing, mining, and construction (2). In China, more than 80 million workers are exposed to occupational noise, and ONIHL remains a major occupational health concern (3, 4).

Baiyin City is a typical resource-based industrial city in China, where occupational noise exposure remains common in mining and related industries. Research on hearing loss among local noise-exposed workers may help clarify the regional burden of occupational noise hazards and provide evidence for targeted prevention and control.

Long-term exposure to workplace noise above 85 dB(A) can cause irreversible auditory damage. In addition to direct mechanical injury to cochlear hair cells, co-exposure to other occupational hazards may exacerbate hearing damage through oxidative stress, vascular injury, and neural toxicity pathways (5).

Noise-induced inner ear damage is typically characterized by temporary or permanent threshold shift. Occupational noise exposure has also been linked to non-auditory outcomes, including sleep disorders, endocrine dysfunction, and cardiovascular disease (6, 7). Because ONIHL is generally irreversible and lacks specific treatment, prevention is essential (8).

Workplace hazards often occur as mixed exposures rather than in isolation. Noise frequently coexists with other harmful agents, and combined exposure may increase the risk of hearing loss. Previous studies have shown that co-exposure to noise and chemical agents can worsen auditory damage (9–11). In coal mining and related industries, workers are commonly exposed to both high-intensity noise and coal dust, yet evidence on their effects on hearing remains limited.

This cross-sectional study examined hearing loss among workers exposed to noise alone or combined noise and coal dust, and explored factors associated with hearing impairment. The findings may support more targeted occupational health prevention and hearing conservation strategies.

## 2 Subjects and methods

### 2.1 Study population and participant selection

This cross-sectional study included workers exposed to occupational noise alone or combined noise and coal dust who underwent occupational health examinations at a designated occupational health institution in Baiyin City, China, in 2023.

Examinations were conducted in accordance with the Technical Specifications for Occupational Health Surveillance (GBZ 188–2019).

**Figure 1:**
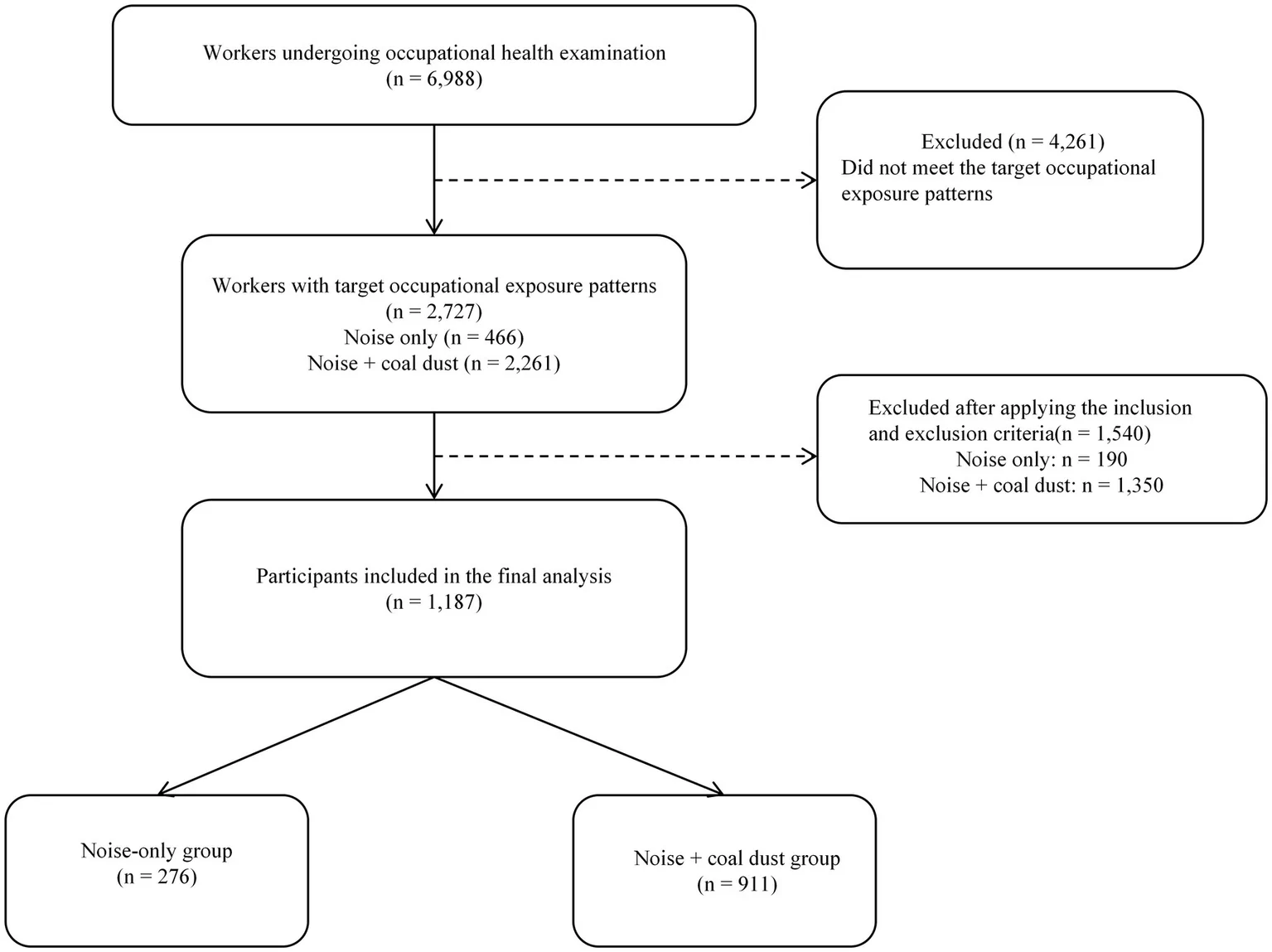
Flow diagram of participant selection.

Eligible participants had at least 3 years of occupational exposure to noise or combined noise and coal dust. Workers with contraindications for noise-exposed work, a history of occupational deafness, or other identifiable causes of hearing loss were excluded. A total of 6,988 workers underwent occupational health examinations during the study period. Of these, 2,727 met the predefined occupational exposure patterns, including 466 workers exposed to noise alone and 2,261 workers exposed to noise plus coal dust. After application of the inclusion and exclusion criteria, 1,540 workers were excluded, leaving 1,187 participants for the final analysis. The final study population comprised 276 workers in the noise-only group and 911 workers in the noise + coal dust group. The participant selection process is shown in Figure 1.

### 2.2 Research methods

#### 2.2.1 Questionnaire survey

Data were collected using a structured questionnaire administered by trained investigators. The questionnaire included demographic characteristics, occupational history, exposure to occupational hazards, medical and medication history, and family history of hereditary hearing loss. All participants provided informed consent, and personal information was kept confidential.

#### 2.2.2 Pure-tone audiometry

Pure-tone audiometry was performed 48 h after participants had left the noise environment to minimize the influence of temporary threshold shift. Before testing, the external ear canal and tympanic membrane were examined by a physician. Audiometric assessors were not provided with participants’ occupational exposure classifications during hearing testing. Audiometric testing was conducted in a soundproof room with background noise below 25 dB(A), using an AS216 audiometer. The audiometer was routinely calibrated in accordance with, and calibration was valid during the study periodAir-conduction thresholds were measured bilaterally at 500, 1000, 2000, 3,000, 4,000, and 6,000 Hz.

#### 2.2.3 Definition of hearing loss

Hearing loss was defined as a hearing threshold >25 dB HL based on the WHO hearing-impairment grading scheme used in the present study, in which hearing levels of 26–40 dB HL were classified as slight/mild hearing impairment. Speech-frequency hearing loss (SFHL) was defined as a binaural average threshold >25 dB HL at 500, 1000, and 2000 Hz, and HFHL was defined using the arithmetic mean of the six measured air-conduction thresholds at 3000, 4000, and 6,000 Hz in both ears.

#### 2.2.4 Blood pressure assessment

Systolic and diastolic blood pressure status were included as potential vascular-related confounding factors. According to the clinical criteria applied during the occupational health examination, systolic blood pressure ≥140 mmHg was classified as abnormal systolic blood pressure, and diastolic blood pressure ≥90 mmHg as abnormal diastolic blood pressure; values below these respective thresholds were classified as normal. The normal category was used as the reference group.

#### 2.2.5 Assessment of occupational noise and coal dust exposure

Occupational noise exposure was assessed using stationary measurements at fixed workstations rather than personal noise dosimetry. Measurements were conducted under normal production conditions in accordance with the Chinese national occupational health standard GBZ/T 189.8-2007, using an SE-402-IS integrating sound level meter (TSI Incorporated, USA). The instrument was within its valid calibration period and was calibrated before measurement. Monitoring points were selected according to job category and workstation. Both steady-state and non-steady-state occupational noise were present during monitoring. No impulsive noise was identified during the monitoring period. The principal occupational noise sources included coal shearers, scraper conveyors, single hydraulic props, articulated roof beams, emulsion pumps, spray pumps, pneumatic rock drills, roof-bolting drills, and pneumatic picks. Because multiple pieces of equipment may operate simultaneously during routine mining activities, the measured noise levels represented the overall noise environment at the corresponding workstation rather than source-specific sound pressure levels generated by individual machines. Noise levels were measured at approximately ear level over three consecutive days. At each monitoring point, three repeated measurements were performed per day, and the mean value was used to characterize the daily noise level at that workstation. Formal homogeneous exposure groups were not established.

Respirable coal dust exposure was assessed using fixed-location sampling under normal production conditions in accordance with GBZ/T 192.2–2007. Samples were collected using FCC30 (Tianyue Incorporated, China) dust samplers at fixed monitoring locations representative of approximately 25 job categories. The sampling flow rate was 20 L/min, and the sampling duration was 15 min per sample. All sampling instruments were within their valid calibration or verification period. Respirable coal dust concentrations were determined gravimetrically, and the 8-h time-weighted average concentration was calculated according to the applicable occupational health standard.

Participants were classified according to occupational hazard assessment as either workers exposed to occupational noise alone or workers jointly exposed to occupational noise and coal dust. However, the coal dust samples collected in the present study were not further characterized for their chemical, mineralogical, microscopic, or metallic composition.

Workers used 3 M 1110 earplugs for hearing protection, and coal-dust-exposed workers used Powecom 1866 V protective masks during routine work and workplace monitoring. However, individual long-term adherence, consistency of correct use, and fit throughout participants’ occupational histories were unavailable.

#### 2.2.6 Quality control

All investigators received standardized training before data collection. Questionnaire data were checked on the same day and entered using double-entry procedures with consistency verification. Audiometric testing was conducted by trained medical personnel under standardized conditions, and all participants were tested 48 h after leaving the noise environment.

### 2.3 Statistical analysis

Statistical analyses were performed using SPSS version 26.0. Continuous variables were assessed for normality and are presented as mean ± standard deviation for approximately normally distributed data and as median (interquartile range) for non-normally distributed data, where applicable. Categorical variables are presented as counts and percentages. Between-group comparisons were performed using the independent-samples *t* test or Mann–Whitney *U* test for continuous variables, as appropriate, and the chi-square test or Fisher’s exact test for categorical variables. For frequency-specific comparisons of audiometric thresholds, the Bonferroni correction was applied to account for multiple testing.

Multivariable binary logistic regression was used to estimate the associations between potential factors and HFHL. Adjusted odds ratios (ORs) and 95% confidence intervals (CIs) were reported. Age and sex were included *a priori* as covariates because the hearing outcomes were defined using measured audiometric thresholds without age- or sex-related correction and because both are established determinants of hearing function. Systolic and diastolic blood pressure status were included as potential vascular-related confounders. The linearity of the logit for continuous covariates was assessed before model interpretation. Multicollinearity among covariates was evaluated using correlation analysis, tolerance values, and variance inflation factors (VIFs). Given the correlation between age and occupational exposure duration, sensitivity analyses were conducted by refitting the multivariable model after separately excluding age and occupational exposure duration to assess the robustness of the estimated association between exposure group and HFHL. Model goodness-of-fit was evaluated using the Hosmer–Lemeshow test. All statistical tests were two-sided, and a *p* value <0.05 was considered statistically significant, except where Bonferroni correction was applied.

## 3 Results

### 3.1 Participant characteristics

A total of 1,187 workers were included in the study, including 276 workers in the noise-only group and 911 workers in the noise + coal dust group (Table 1). Participants in the noise-only group were older than those in the noise + coal dust group (46.96 ± 7.03 vs. 43.90 ± 8.32 years, *p* < 0.001) and had a longer occupational exposure duration (23.35 ± 8.65 vs. 18.85 ± 9.69 years, *p* < 0.001). The proportion of men was higher in the noise + coal dust group than in the noise-only group (92.5% vs. 79.3%, *p* < 0.001). The prevalence of HFHL was also higher in the noise + coal dust group 46.8 . The prevalence of abnormal diastolic blood pressure differed between the two groups 26.2 , whereas no significant between-group differences were observed for BMI or abnormal systolic blood pressure.

**Table 1:** Characteristics of participants according to occupational exposure group.

Variables	Total (*n* = 1,187)	Noise only (*n* = 276)	Noise + coal dust (*n* = 911)	*p* value
Age, years	44.61 ± 8.14	46.96 ± 7.03	43.90 ± 8.32	<0.001
Male, *n* (%)	1,062 (89.5)	219 (79.3)	843 (92.5)	<0.001
Occupational exposure duration, years	19.89 ± 9.64	23.35 ± 8.65	18.85 ± 9.69	<0.001
BMI, kg/m^2^	24.78 ± 3.19	24.62 ± 3.26	24.83 ± 3.16	0.318
Abnormal systolic blood pressure, *n* (%)	179 (15.1)	47 (17.0)	132 (14.5)	0.302
Abnormal diastolic blood pressure, *n* (%)	332 (28.0)	93 (33.7)	239 (26.2)	0.016
HFHL, *n* (%)	524 (44.1)	98 (35.5)	426 (46.8)	0.001

### 3.2 Occupational hazard measurements

#### 3.2.1 Noise

Workplace noise was measured at fixed sites over 3 consecutive days in both exposure groups, and the 8-h-equivalent workstation noise levels ranged from 76.6 to 86.2 dB(A). Mean noise levels were similar between groups across all 3 days: 82.37 vs. 82.74 dB(A) on day 1, 80.65 vs. 81.67 dB(A) on day 2, and 83.82 vs. 83.17 dB(A) on day 3 for the noise-only and combined exposure groups, respectively. None of these differences were statistically significant (all *p* > 0.05).

#### 3.2.2 Coal dust

Workers in the combined exposure group were exposed to both noise and coal dust. Respirable coal dust was measured at 30 sampling sites during work shifts. The 8-h time-weighted average concentration ranged from 0.10 to 4.05 mg/m^3^, with a mean of 1.33 ± 0.79 mg/m^3^, and all measurements were below the occupational exposure limit.

### 3.3 Hearing loss among workers

#### 3.3.1 Speech-frequency hearing loss

A total of 21 cases of SFHL were identified, indicating a low overall prevalence. No clear differences in SFHL were observed by sex, age, years of exposure, or occupational exposure group. The prevalence of SFHL was 1.45% in the noise-only group and 1.87% in the combined noise–coal dust exposure group. Because of the small number of SFHL cases, these results are presented descriptively (Table 1).

#### 3.3.2 High-frequency hearing loss

HFHL was more common than SFHL. HFHL prevalence differed significantly by sex, age, years of exposure, and occupational exposure group, with higher rates observed in men, older workers, and those with longer exposure duration. HFHL prevalence was 35.51% in the noise-only group and 46.76% in the combined noise–coal dust exposure group. Most physiological and biochemical indicators were not associated with HFHL, although workers with abnormal systolic or diastolic blood pressure had a higher prevalence of HFHL (Table 2).

**Table 2:** Distribution characteristics of hearing loss.

Variables	ALL	SFHL			HFHL		
		Number (*n*, %)	*χ* ^2^	*p*	Number (*n*, %)	*χ* ^2^	*p*
Sex			0.023	*p* = 0.616		44.884	*p* < 0.001
Male	1,062	19 (1.79%)			504 (47.46%)		
Female	125	2 (1.60%)			20 (16.0%)		
Age (years)			2.603	*p* = 0.272		50.160	*p* < 0.001
~35	206	1 (0.49%)			47 (22.82%)		
35–50	672	15 (2.23%)			312 (46.43%)		
50–	309	5 (1.62%)			165 (53.40%)		
Years of exposure to hazards (years)			4.570	*p* = 0.096		16.775	*p* < 0.001
~15	415	4 (0.96%)			150 (36.14%)		
15–30	557	15 (2.69%)			267 (47.94%)		
30–	215	2 (0.93%)			107 (49.77%)		
Risk factors			0.212	*p* = 0.439		10.882	*p* < 0.001
Noise	276	4 (1.45%)			98 (35.51%)		
Noise and dust	911	17 (1.87%)			426 (46.76%)		
Diastolic blood pressure			0.305	*p* = 0.368		3.063	0.046
Normal	855	14 (1.64%)			364 (42.57%)		
Abnormal	322	7 (2.11%)			160 (48.19%)		
Systolic blood pressure			0.011	*p* = 0.607		5.988	0.009
Normal	1,008	18 (1.79%)			430 (42.66%)		
Abnormal	179	3 (1.68%)			94 (52.51%)		
ECG			0.031	*p* = 0.513		0.138	0.380
Normal	868	15 (1.73%)			386 (44.47%)		
Abnormal	319	6 (1.88%)			138 (43.26%)		
Hemoglobin			0.100	*p* = 0.517		0.012	0.486
Normal	987	18 (1.82%)			435 (44.07%)		
Abnormal	200	3 (1.50%)			89 (44.50%)		
Red blood cell count			0.065	*p* = 0.525		0.575	0.246
Normal	934	17 (1.82%)			407 (43.58%)		
Abnormal	253	4 (1.58%)			117 (46.25%)		
White blood cell count			2.162	*p* = 0.130		0.398	0.299
Normal	1,078	21 (1.95%)			479 (44.43%)		
Abnormal	109	0 (0.00%)			45 (41.28%)		
Platelet count			1.401	*p* = 0.261		0.186	0.377
Normal	1,114	21 (1.89%)			490 (43.99%)		
Abnormal	73	0 (0.00%)			34 (46.58%)		
Urinary protein			0.348	*p* = 0.711		0.418	*p* = 0.343
Normal	1,168	21 (1.80%)			517 (44.26%)		
Abnormal	19	0 (0.00%)			7 (36.84%)		
Glucose in urine			3.006	*p* = 0.194		1.802	0.147
Normal	1,175	20 (1.70%)			521 (44.34%)		
Abnormal	12	1 (8.33%)			3 (25.00%)		
Urine occult blood			0.026	*p* = 0.672		1.113	0.117
Normal	1,121	20 (1.78%)			499 (44.51%)		
Abnormal	66	1 (1.52%)			25 (37.88%)		
White blood cells in urine			0.688	*p* = 0.511		1.257	0.170
Normal	1,150	21 (1.83%)			511 (44.43%)		
Abnormal	37	0 (0.00%)			13 (35.14%)		
pH			0.567	*p* = 0.305		2.715	0.058
Normal	978	16 (1.64%)			421 (43.05%)		
Abnormal	209	5 (2.39%)			103 (49.28%)		

### 3.4 Multivariable logistic regression analysis

In the multivariable logistic regression analysis, age, sex, occupational exposure duration, and occupational exposure pattern were significantly associated with HFHL after adjustment for the other covariates included in the model (Table 3). Older age was associated with higher odds of HFHL (OR = 1.073, 95% CI: 1.049–1.098, *p* < 0.001). Men had higher odds of HFHL than women (OR = 3.838, 95% CI: 2.313–6.371, *p* < 0.001). Occupational exposure duration showed a small inverse association with HFHL (OR = 0.981, 95% CI: 0.964–1.000, *p* = 0.045). Workers exposed to both noise and coal dust had higher odds of HFHL than those exposed to noise alone (OR = 1.620, 95% CI: 1.198–2.190, *p* = 0.002). Neither systolic nor diastolic blood pressure status was significantly associated with HFHL (OR = 1.180, 95% CI: 0.794–1.755, *p* = 0.413 and OR = 0.944, 95% CI: 0.685–1.300, *p* = 0.723, respectively). BMI was also not significantly associated with HFHL (OR = 0.995, 95% CI: 0.957–1.035, *p* = 0.813).

**Table 3:** Multivariable logistic regression analysis of factors associated with HFH.

Variables	*β*	S.E.	Wald	P	OR	95% CI
Ages (years)	0.070	0.012	37.538	0.000	1.071	1.049–1.098
Years of exposure to hazards (years)	−0.019	0.009	4.002	0.045	0.981	0.964–1.000
BMI	0.005	0.020	0.056	0.813	1.005	0.966–1.045
Sex						
Female					1.000(Ref.)	
Male	1.345	0.258	27.076	0.000	3.838	2.313–6.371
Occupational exposure pattern						
Noise					1.000(Ref.)	
Noise and dust	0.482	0.154	9.844	0.002	1.620	1.198–2.190
Diastolic blood pressure						
Normal					1.000(Ref.)	
Abnormal	−0.058	0.164	0.126	0.723	0.944	0.685–1.300
Systolic blood pressure						
Normal					1.000(Ref.)	
Abnormal	0.166	0.202	0.669	0.413	1.180	0.794–1.755

### 3.5 Model diagnostics and sensitivity analyses

Age and occupational exposure duration were positively correlated (Pearson’s *r* = 0.732, *p* < 0.001). However, the corresponding VIF values were 2.178 and 2.172, respectively, suggesting no substantial multicollinearity.

In sensitivity analyses, the association between noise + coal dust exposure and HFHL remained statistically significant after exclusion of age (OR = 1.557, 95% CI: 1.156–2.097, *p* = 0.004) and after exclusion of occupational exposure duration (OR = 1.676, 95% CI: 1.242–2.261, *p* = 0.001). These estimates were similar to that obtained in the fully adjusted model (OR = 1.620, 95% CI: 1.198–2.190), supporting the robustness of the association between occupational exposure pattern and HFHL. In contrast, the direction of the association between occupational exposure duration and HFHL changed after age was excluded from the model, indicating that the estimate for occupational exposure duration was sensitive to adjustment for age. The Hosmer–Lemeshow goodness-of-fit test showed no evidence of poor model fit χ2=8.249,p=0.410 .

### 3.6 Frequency-specific hearing loss

**Figure 2:**
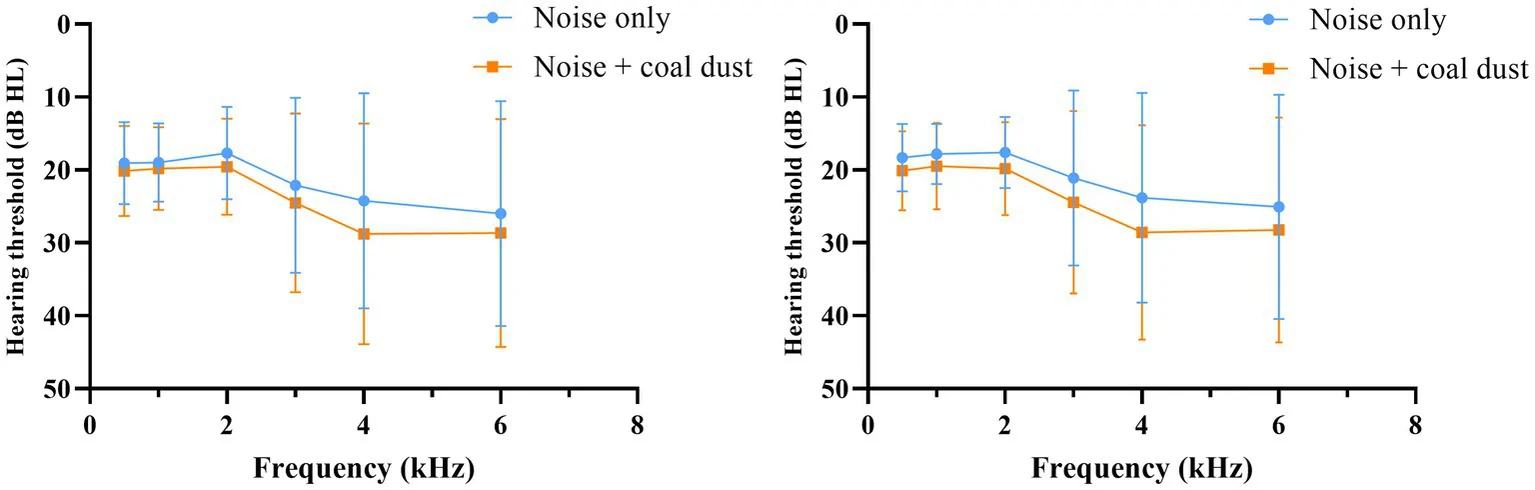
Audiograms of the left and right ears.

Hearing abnormalities in both exposure groups were predominantly observed at high frequencies, particularly at 3,000, 4,000, and 6,000 Hz. No significant between-group differences were observed at 500, 1,000 2,000, or 3,000 Hz. Before correction for multiple comparisons, differences were observed at some higher frequencies; however, after Bonferroni correction, only the between-group differences at 4000 Hz in both ears remained statistically significant. Workers exposed to both noise and coal dust had a higher prevalence of abnormal thresholds at 4000 Hz than those exposed to noise alone (Table 4 and Figure 2). These findings indicate that the most consistent frequency-specific difference between the two exposure groups occurred at 4000 Hz.

**Table 4:** Frequency-specific abnormal hearing thresholds according to occupational exposure group.

Variables	Total	Noise only (*N* = 276)	Noise + dust (*N* = 911)	*χ* ^2^	*p* value
L-Frequency (Hz)					
500	20	3 (1.09%)	17 (1.87%)	0.776	0.280
1,000	18	3 (1.09%)	15 (1.65%)	0.778	0.367
2,000	23	5 (1.81%)	18 (1.98%)	0.030	0.547
3,000	343	72 (26.09%)	271 (29.75%)	1.381	0.135
4,000	495	87 (31.52%)	408 (44.79%)	15.330	*p* < 0.001
6,000	544	110 (39.86%)	434 (47.64%)	5.171	0.014
R-Frequency (Hz)					
500	20	5 (1.81%)	15 (1.65%)	0.035	0.513
1,000	21	6 (2.17%)	15 (1.65%)	0.339	0.359
2,000	25	7 (2.54%)	18 (1.98%)	0.323	0.358
3,000	335	73 (26.45%)	262 (28.76%)	0.558	0.252
4,000	496	91 (32.97%)	405 (44.46%)	11.448	*p* < 0.001
6,000	527	115 (41.67%)	412 (45.23%)	1.086	0.165

## 4 Discussion

ONIHL, especially HFHL, is irreversible and remains a major target for prevention because effective treatment options are limited (12). In this study, workers in the combined noise–coal dust exposure group had higher prevalences of HFHL than those in the noise-only group, suggesting that compared with noise exposure alone, combined exposure to noise and coal dust was independently associated with higher odds of high-frequency hearing loss. These findings suggest that coal dust co-exposure may contribute to an additional adverse association with hearing among noise-exposed workers, although causal or synergistic effects cannot be established from this cross-sectional study. This finding is consistent with the elevated burden of hearing impairment reported in mining populations exposed to complex occupational hazards (13).

Pure-tone audiometry showed that hearing abnormalities in both groups were concentrated at high frequencies, most evident at 4000 Hz bilaterally, which is consistent with the typical audiometric pattern of HFHL (14, 15). This pattern supports the view that occupational noise-related damage first affects the high-frequency range and may later extend to speech frequencies as exposure continues.

HFHL was substantially more common than SFHL in the study population, indicating that high-frequency impairment may represent an earlier and more sensitive marker of occupational hearing damage (16). By contrast, SFHL was uncommon and showed no clear association with sex, years of exposure, or occupational exposure pattern (17). But in this study, only 21 participants met the definition of SFHL, resulting in limited statistical power for evaluating between-group differences. Therefore, the absence of a statistically significant association should not be interpreted as evidence that combined exposure has no association with speech-frequency hearing impairment.

HFHL was more common than SFHL in this study, and its prevalence increased with age and duration of occupational exposure. Age and occupational exposure duration showed closely related but distinct associations with HFHL. Older age was associated with higher odds of HFHL in the fully adjusted model (OR = 1.073, 95% CI: 1.049–1.098), consistent with the age-related increase in HFHL prevalence observed in the stratified analyses. Occupational exposure duration, however, showed a small inverse association with HFHL in the fully adjusted model (OR = 0.981, 95% CI: 0.964–1.000). This finding should be interpreted cautiously because age and occupational exposure duration were strongly correlated (Pearson’s *r* = 0.732). Although collinearity diagnostics did not indicate substantial multicollinearity, sensitivity analyses showed that the coefficient for occupational exposure duration changed direction after age was excluded from the model (OR = 1.023, 95% CI: 1.010–1.036), whereas the positive association between age and HFHL persisted after occupational exposure duration was excluded (OR = 1.056, 95% CI: 1.039–1.073). These findings suggest that the estimate for occupational exposure duration was sensitive to mutual adjustment with age and should therefore not be interpreted as evidence of a protective effect of longer occupational exposure. Although cumulative occupational noise exposure has been associated with hearing impairment in previous studies (18), the present cross-sectional data do not allow a clear independent cumulative effect of exposure duration to be established.

HFHL was also substantially more common in men than in women. Men had a 3.84-fold higher risk of HFHL, consistent with previous studies (18, 19). This difference may be partly explained by occupational role patterns, as men are more likely to work in front-line positions in high-noise industries such as mining, heavy industry, and construction (20). Sex differences in hearing protection behaviors may also contribute (21). In addition, estrogen may have neuroprotective and antioxidant effects that reduce noise-related damage to cochlear hair cells (22).

Workers exposed to both noise and coal dust had a higher prevalence of HFHL than those exposed to noise alone, and this association remained significant after adjustment for potential confounders. Compared with noise-only exposure, combined noise–coal dust exposure was associated with a 1.62-fold higher risk of HFHL. This is consistent with previous studies suggesting that mixed occupational exposures may have further association with hearing loss among mine workers (23, 24). Given the cross-sectional design, temporality between occupational exposure and hearing impairment could not be established, and the observed associations should not be interpreted as causal effects.

A possible explanation is that coal dust is a complex mixture containing components such as quartz, pyrite, and heavy metals, which may contribute to cochlear injury through oxidative stress and inflammatory pathways (25, 26). Excess reactive oxygen species may also induce mitochondrial dysfunction and apoptosis in hair cells and spiral ganglion neurons, leading to irreversible auditory damage because of the limited regenerative capacity of mammalian inner ear cells (27, 28). These mechanisms may help explain the effect of combined noise and coal dust exposure on HFHL.

HFHL may also be associated with broader health effects beyond hearing impairment. Persistent hearing loss and tinnitus can adversely affect mental wellbeing and sleep, and occupational noise exposure has also been linked to hypertension (29, 30). In this study, hypertension was more common among workers with HFHL in unadjusted analyses, but the association was not significant in the multivariable model, suggesting possible confounding by age, years of exposure, or related factors.

Several limitations should be considered when interpreting the present findings. First, the cross-sectional design precludes determination of temporal or causal relationships between occupational exposure patterns and HFHL; therefore, the observed associations should not be interpreted as evidence that coal dust exposure causes or aggravates hearing loss. In addition, exposure classification was based on occupational exposure patterns rather than detailed individual quantitative estimates of cumulative noise and coal dust exposure, which may have resulted in exposure misclassification and limited assessment of dose–response relationships. Third, participants were workers undergoing occupational health examinations at a single institution in Baiyin City, and further selection occurred according to predefined eligibility criteria; thus, selection bias, including a potential healthy-worker effect and underrepresentation of workers with more severe hearing impairment, cannot be excluded, and the generalizability of the findings to other occupational populations may be limited. And HFHL was defined using measured air-conduction thresholds at 3000, 4000, and 6,000 Hz without bone-conduction testing. Therefore, the outcome represents an audiometric high-frequency hearing abnormality rather than a clinical diagnosis of occupational noise-induced hearing loss, and conductive components of hearing impairment could not be completely distinguished. Although age and sex were included in the multivariable model because the audiometric thresholds were not corrected for these factors, residual confounding from unmeasured or incompletely measured factors, such as non-occupational noise exposure, lifestyle characteristics, comorbidities, and ototoxic exposures, may remain. Finally, age and occupational exposure duration were strongly correlated. Although variance inflation factors did not indicate substantial multicollinearity and the association between occupational exposure pattern and HFHL remained stable in sensitivity analyses, the direction of the coefficient for occupational exposure duration changed after age was removed from the model. Accordingly, the estimated association for occupational exposure duration should be interpreted cautiously. Prospective multicenter studies incorporating quantitative individual exposure assessment, more comprehensive audiological evaluation, and additional potential confounders are needed to confirm these findings and clarify the temporal and dose–response relationships.

## 5 Conclusion

Mining remains a high-risk industry for occupational noise-induced hearing loss because workers are commonly exposed to multiple hazards, including noise and coal dust. In this study, hearing impairment was predominantly high-frequency, was more common with longer exposure duration, and was more frequent in men. Hearing abnormalities were concentrated around 4,000 Hz, consistent with the typical pattern of occupational noise-related damage. In this cross-sectional sample, workers classified as jointly exposed to occupational noise and coal dust had higher adjusted odds of HFHL than workers classified as exposed to occupational noise alone. Because individual cumulative exposure, several potentially important confounders, workplace-level clustering, and formal exposure interaction were not fully assessed, these findings do not establish a causal or synergistic effect. The findings highlight the importance of integrated hearing conservation, occupational health surveillance, and early preventive interventions for workers with combined occupational exposures.

## Data Availability

The occupational health examination data are managed by the relevant disease-control institution and are not publicly available because of confidentiality and institutional data-governance requirements. Requests for access to de-identified data may be considered in accordance with the policies and approval procedures of the data-holding institution. After obtaining third-party permission, data analyzed during the current study are available from the corresponding author (ruany1203@163.com) on reasonable request.
